# The (in)visibility of women in science

**DOI:** 10.3325/cmj.2026.67.1

**Published:** 2026-02

**Authors:** Svjetlana Kalanj Bognar, Klementina Fon, Tacer, Ivana Rosenzweig

**Affiliations:** 1 Editor-in-Chief, *Croatian Medical Journal*; 2 Zagreb University School of Medicine, Zagreb, Croatia; *svjetlana.kalanj.bognar@cmj.hr*; 3 School of Veterinary Medicine, Texas Tech University, Amarillo, TX, USA; 4 Texas Center for Comparative Cancer Research (TC3R), Amarillo, TX, USA; 5 Editor-in-Chief, *Slovenian Veterinary Journal*; 6 Sleep and Brain Plasticity Centre, King's College London, London, UK; 7 Institute of Psychiatry, Psychology & Neuroscience, King's College London, London, UK

Each year, the brief stretch between February 11, the International Day of Women and Girls in Science, and March 8, International Women's Day, offers more than two commemorative dates (1-3). It offers a mirror. It asks whether science has truly learned to see all those who sustain it. Science is often described as a universal language: rigorous, self-correcting, indifferent to nationality, class, or gender. At its best, it is exactly that: a disciplined search for truth, animated by curiosity, imagination, skepticism, courage, and persistence. Yet, science has always been practiced in human institutions, and human institutions do not distribute recognition evenly. On International Women's Day, this is worth stating plainly: women have always helped build science, but too often they have not been seen proportionally to their contribution.

Despite changes enabling women to pursue education and scientific, particularly in recent decades, the contributions of female scientists are still marginalized or inadequately acknowledged. In the era of exceptional global growth of scientific knowledge, scientific community has to do more to ensure equal educational opportunities and to include talented individuals with no other privileges besides their competence and potential. For many centuries, higher education was an unrealistic dream for the majority of women. Even as more civil rights and justice are being provided to women, the proportion of women working in science or being recognized for their work has been lagging behind their male colleagues. One of the clearest mirrors of the imbalance is the architecture of prestige. The Nobel Prize is not a perfect proxy for scientific worth, but it is a powerful symbol of who history chooses to remember. The scientific prize has been awarded since 1901 to 990 individuals, of which only 67 are women, with Marie Curie remaining the only woman honored twice (4,5)! Fewer than half of those awards given to women were in STEM fields – chemistry, physics, and physiology or medicine. From 2021 to 2025, out of 57 Nobel Prizes in all scientific categories, only 10 were awarded to women (4 in physics, chemistry, and physiology or medicine), which indicates persistent under-recognition of female scientists’ achievements (5). All these numbers do not tell us that women lack brilliance; they tell us that brilliance has never been equally noticed, rewarded, or narrated.

There has been progress, and it should be acknowledged without hesitation. More women are entering higher education, earning doctoral degrees, leading research groups, and transforming science. According to Eurostat, women now account for 52% of those employed in science and technology. Yet, they still represent less than one third of the world's researchers. The story, then, is not one of absence. It is one of attrition, friction, and insufficient recognition (2,6,7). Part of the problem lies in how scientific merit has been imagined. Discovery is still too easily attributed to a single heroic mind, when in reality science advances through collective effort: through method development, difficult experimental labor, clinical observation, collaboration, mentorship, and persistence. Some of the most important work in science is not loud. It is precise. It is cumulative. It makes a breakthrough possible. When recognition systems prefer the myth of genius to the full ecology of discovery, they misrecognize the work itself. Women have too often been placed in the background of this picture – present, essential, and insufficiently named. The historical oversight of diverse contributions to scientific discoveries also reflects limitations in how merit is defined and assessed, and disproportionately affects women.

A disparity between the number of women working in specific scientific fields and those in leadership roles, or receiving support for career advancement, is also observed in medical and veterinary professions. Several studies report on worrisome underrepresentation of women as editors-in-chief in the most prominent medical journals, revealing that only 21% of more than 400 analyzed medical journals had female editors (8,9). When editorial leadership remains imbalanced, visibility remains imbalanced too. Journals do not simply report science; they help decide what counts as authoritative science. They determine which questions are amplified, which methods are legitimized, whose voices are commissioned to interpret the field, and which names become familiar to readers. In medicine, this has practical consequences: research priorities, clinical assumptions, and public health agendas are all shaped by those whose expertise is treated as central rather than supplementary. Science becomes better not when standards are lowered, but when the playing field is no longer tilted.

Visibility, therefore, must be understood as a daily practice, not an annual ceremony. It begins with education, but it does not end there. It lives in hiring committees that look beyond inherited prototypes of authority. It lives in promotion systems that value mentoring, collaboration, translational work, and team science instead of rewarding only the loudest or most conventional career trajectories. It lives in fair credit for ideas, methods, experimental labor, and leadership. It lives in conference programs, prize nominations, reviewer pools, editorial boards, and invited commentaries. For journals especially, it lives in the willingness to ask uncomfortable questions: Whose work do we treat as seminal? Whom do we routinely invite to speak for the field? Whose excellence have we normalized, and whose excellence do we still describe as exceptional? For editors, the responsibility is immediate. We can widen reviewer pools, diversify editorial leadership, and commission women not only on “women's issues” but across the full range of scientific and clinical debate. We can examine citation practices, invited authorship, and speaking opportunities. We can resist the old reflex of equating authority with familiarity when familiarity itself has been shaped by exclusion. None of this requires tokenism. It requires attention, seriousness, and institutional memory. It requires us to understand that fairness is not peripheral to excellence; it is one of its conditions.

Visibility in science is not a cosmetic matter. It is not about applause, branding, or symbolic inclusion. When women are under-recognized, science does not merely commit a moral error; it distorts its own record. And when that distortion is repeated often enough, it begins to feel natural. It shapes the stories institutions tell about excellence, authority, and intellectual inheritance.

Still, this is not a pessimistic story. It is a demanding one. The point is not to rehearse grievances, but to widen possibilities. Every time a young woman sees a scientist whose authority is unqualified, whose name is cited without surprise, whose leadership is ordinary rather than novel, the horizon of the possible expands. And every time an institution chooses fairness over familiarity, it makes science more truthful about who creates knowledge. The task before us is therefore larger than correcting a historical omission. It is to refuse a diminished picture of science itself. Science advances when talent is recognized early, supported consistently, credited fairly, and remembered accurately. It advances when women are not treated as welcome exceptions but as indispensable authors of discovery.

Apparently, gender equality requires not only profound analysis but our continuous attention, advocacy, and action. These are the very goals of the initiative International Day of Women and Girls in Science, and proclaimed as one of the main topics of the 2026 International Women’s Day – right, action and justice for all women and girls (3). On this International Women's Day, we should say it clearly: the visibility of women in science is not a peripheral concern. It is central to excellence, central to justice, and central to the future credibility of science. In addition, the International Day of Women and Girls in Science and International Women's Day remind us not only of gender equality but of equal opportunities in education and social justice for everyone. As John Lennon once said, “…Imagine all the people sharing all the world...”. We may add, “Imagine the mastery of women in science being visible in all the world.”
